# The Future of Artificial Intelligence in Mental Health Nursing Practice: An Integrative Review

**DOI:** 10.1111/inm.70003

**Published:** 2025-01-23

**Authors:** Lucian H. Milasan, Daniel Scott‐Purdy

**Affiliations:** ^1^ Institute of Health and Allied Professions Nottingham Trent University Nottingham UK

**Keywords:** artificial intelligence (AI), mental health nursing, psychiatric nursing, psychiatry

## Abstract

Artificial intelligence (AI) has been increasingly used in delivering mental healthcare worldwide. Within this context, the traditional role of mental health nurses has been changed and challenged by AI‐powered cutting‐edge technologies emerging in clinical practice. The aim of this integrative review is to identify and synthesise the evidence of AI‐based applications with relevance for, and potential to enhance, mental health nursing practice. Five electronic databases (CINAHL, PubMed, PsycINFO, Web of Science and Scopus) were systematically searched. Seventy‐eight studies were identified, critically appraised and synthesised following a comprehensive integrative approach. We found that AI applications with potential use in mental health nursing vary widely from machine learning algorithms to natural language processing, digital phenotyping, computer vision and conversational agents for assessing, diagnosing and treating mental health challenges. Five overarching themes were identified: assessment, identification, prediction, optimisation and perception reflecting the multiple levels of embedding AI‐driven technologies in mental health nursing practice, and how patients and staff perceive the use of AI in clinical settings. We concluded that AI‐driven technologies hold great potential for enhancing mental health nursing practice. However, humanistic approaches to mental healthcare may pose some challenges to effectively incorporating AI into mental health nursing. Meaningful conversations between mental health nurses, service users and AI developers should take place to shaping the co‐creation of AI technologies to enhance care in a way that promotes person‐centredness, empowerment and active participation.

## 1 Introduction

Artificial intelligence (AI) has been increasingly present in our society whether visibly present or hidden in every day digital practice, and the healthcare sector is no exception. There are strong indications that AI has the potential to enhance the quality of care, patient experience and staff satisfaction (Davenport and Kalakota 2019). Furthermore, AI can contribute to optimising health services and resources by reducing costs, improving administrative processes, and supporting the decision‐making process within health organisations (Dicuonzo et al. 2023).

Mental health services have witnessed a growing interest from AI developers designing a range of interventions to support mental wellbeing. For example, chatbot‐based mental health apps and virtual therapists for depression and anxiety (Ahmed et al. 2023; Anmella et al. 2023; Haque and Rubya 2023), AI‐powered VR therapy in treating phobias through exposure therapy (Gomes et al. 2023), AI‐assisted diagnosis and treatment (Shatte, Hutchinson, and Teague 2019), and personalised care plans and assessments aided by AI predictive algorithms (Koutsouleris et al. 2022).

From a plethora of definitions formulated since the concept was advanced in the 1950s, we adopted McGrow's (2019) definition of AI as ‘computer systems able to complete tasks that typically require human intelligence, such as visual perception, speech recognition, decision‐making, and/or language translation’ (p. 46). In other words, AI is a collection of technologies able to learn independently, create and make decisions and inferences similar to, or even better than, humans without relying on explicit programming to perform such tasks (Robert 2019). Machine learning (ML), deep learning (DL), natural language processing (NLP), artificial neural networks, digital phenotyping, computer vision and speech recognition are only a few subsets of AI (Gerich et al. 2022) whose functionality and applicability to nursing will be discussed in this review.

AI's revolutionising role in mental healthcare is recognised by WHO (2023a) in terms of supporting effective planning of mental health services, identifying and monitoring mental wellbeing. This is facilitated by the digitalisation of health records and opportunities offered by AI to process high volumes of data often unstructured (Yang et al. 2022).

However, some authors describe the use of AI as being more modest in mental healthcare compared to physical health specialities potentially due to the psychosocial complexities and subjectivity of mental distress (Jin et al. 2023). A considerable body of literature also draws attention to ethical challenges related to embedding AI in mental healthcare, such as data privacy and security, algorithmic bias in diagnosing and treating mental health conditions, and AI opacity leading to limited comprehension of decision‐making processes (Rubeis 2022; Warrier, Warrier, and Khandelwal 2023). Some other authors highlight the humanistic nature of therapeutic relationships in mental healthcare that would make the incorporation of machine‐led tasks more challenging compared to other health settings (Sebri et al. 2020). This is a critical aspect of mental health nursing in which the quality of the therapeutic relationship and recovery outcomes for patients are significantly affected by humanistic care (Kogstad, Ekeland, and Hummelvoll 2011). The challenges related to implementing AI in clinical practice are not limited to mental health nursing. The entire healthcare sector is facing issues such as data quality and security, bias and discrimination, for example, perpetuating structural racism, digital inequities, lack of standards and regulatory frameworks, and skills gap in the workforce concerning the understanding and utilisation of AI technologies (Kelly et al. 2019). Furthermore, patient and staff acceptance of AI‐driven solutions continue to be problematic for successfully adopting AI technologies in healthcare despite generally positive attitudes towards AI innovation (Robertson et al. 2023; Scott, Carter, and Coiera 2021).

Regardless of the scepticism and divergences of opinion concerning the future of AI in mental health, advanced technologies continue to transform the sector. It is only a matter of time until the global scale of mental health conditions and high demand for psychiatric and therapeutic services will require solutions that cannot be provided solely by traditional models of mental healthcare (Olawade et al. 2024).

For this reason, it is important to explore the potential of AI‐driven approaches to different areas of mental healthcare. Up to date, this topic has been reviewed rather generically as ‘artificial intelligence in mental health’. However, a closer look at reviews of this topic reveals diagnosis as an overarching theme. For example, Graham et al. (2019) focus on AI's potential to help mental health practitioners diagnose ‘mental illnesses’ more objectively than the current screening and diagnostic approaches. Similarly, Mittal et al.'s (2023) review of the literature on the use of AI in mental healthcare concluded that this technology has the potential to identify patterns and symptoms of various ‘mental disorders’, leading to a more effective detection and personalised treatments. The use of AI in diagnosing ‘mental illnesses’ is also central to Zucchetti et al.'s (2024) review drawing on instrumental techniques such as neuroimaging and neurophysiology combined with different subsets of AI. Other studies seem to be focused on specific AI‐based interventions in mental health, for example, conversational agents (Li et al. 2023), machine learning and deep learning (Iyortsuun et al. 2023), and natural language processing (Zhang et al. 2022). While informative, such resources provide limited knowledge specific to the holistic role of a mental health nurse beyond the bio‐medical model, and how mental health nursing may be affected by embedding AI in this sector in the future, which is covered in our review.

Some of these limitations are partly addressed in reviews of AI in nursing practice (Mahmoudi and Moradi 2024; O'Connor et al. 2022; Pailaha 2023; Ruksakulpiwat et al. 2024). However, references to mental health nursing are lacking or are underrepresented in this literature compared to general nursing. For example, a recent integrative review conducted by Kikuchi (2020) synthesised seventeen nursing studies incorporating AI of which only two from mental health nursing. Based on this low number of studies, it is difficult to translate the findings to mental health nursing which is a field of practice with own particularities and challenges, as acknowledged in this article.

Furthermore, the existing literature on the transformative impact of AI on mental health nursing is methodologically and conceptually limited. For instance, Nashwan, Gharib, and Alhadidi's (2023) study is guided by a methodology insufficiently transparent to support replicability of the search strategy, compared to the systematic approach we adopted in our review. While their results hint to some key aspects of mental health practice and education, their applicability to mental health nursing is not entirely clear potentially because of the generic selection of articles on AI and mental health. Filtering the literature in line with specific tasks performed by mental health nurses, for example, mental health assessments, care planning and implementation, crisis intervention, administration of psychiatric medication, observations and monitoring, that guided our review would provide more targeted and helpful insights for the mental health nursing sector.

We aim to address this knowledge gap by employing a rigorous and replicable integrative methodology to comprehensively identify and synthesise the current evidence of AI‐driven applications with relevance for, and potential to enhance, mental health nursing practice. We translated this aim into a research question that guided the process from searching the relevant literature to analysing and critically discussing data extracted from the selected articles: What potential does Artificial Intelligence (AI) hold in enhancing mental health nursing practice?

## 2 Method

An integrative review framework (Torraco 2005) was adopted to explore the potential of AI in enhancing mental health nursing practice to capture various research designs. The rationale behind this choice was to provide a diversity of insights into the topic under investigation for enhanced comprehension (Dhollande et al. 2021). A systematic review approach relying exclusively on either quantitative, for example, experimental, or qualitative research was initially considered, but we decided that would limit the scope of our research (Owens 2021). Our decision was hindered by the nebulous terminology around literature reviews with some authors supporting mixed‐method or integrative systematic reviews (Pollock and Berge 2018). Despite categorising our review as integrative, it is systematic in the way the search strategy and plan for data collection, extraction and analysis were devised prior to conducting the research, as documented in the review protocol (PROSPERO Ref. CRD42021234843).

Our approach to reviewing the literature aligns with Torraco's (2005) definition of integrative review as a ‘form of research that reviews, critiques, and synthesises representative literature on a topic in an integrated way such that new frameworks and perspectives on the topic are generated’ (p. 356). For consistency, our review was structured around the checklist for organising and writing an integrative literature review provided by the same author (Torraco, 2005, 365).

We systematically searched five electronic databases (CINAHL, PubMed, Web of Science, PsycInfo and Scopus) between January and April 2024. Multiple databases were considered to cover a range of subjects in mental healthcare such as nursing, medicine and psychology. The searches were conducted independently by the two authors (93% agreement, 0.84 Kappa statistic). Disagreements were addressed in the review meetings between the two authors specialised in mental health and digital technologies, respectively. Key terms such as ‘artificial intelligence’, ‘AI’, ‘machine learning’, ‘deep learning’, ‘neural networks’ and ‘mental health nursing’, along with synonyms, were combined in various syntaxes using Boolean operators (Table 1).

**TABLE 1:** Search syntax.

**S1** ‘artificial intelligence’ OR ‘AI’ OR ‘A.I.’ OR ‘generative AI’ OR ‘cognitive comput*’ OR ‘machine learning’ OR ‘algorithm*’ OR ‘deep learning’ OR ‘reinforcement learning’ OR ‘supervised learning’ OR ‘transfer learning’ OR ‘black box’ OR ‘big data’ OR ‘data mining’ OR ‘neural network*’ OR ‘speech recognition’ OR ‘expert system*’ OR ‘natural language processing’ OR ‘NLP’ OR ‘large language model*’ OR ‘predictive analytics’ OR ‘prescriptive analytics’ OR ‘sentiment analysis’ OR ‘chatbot*’ OR ‘voice recognition’ OR ‘robot*’ OR ‘digital phenotyp*’
AND
**S2** ‘mental health nurs*’ OR ‘psychiatric nurs*’ OR ‘mental healthcare’ OR ‘mental health care’ OR ‘psychiatric care’ OR ‘community psychiatr*’ OR ‘forensic nurs*’

The search strategy was piloted independently by the two authors on CINAHL to ensure uniformity of the search process. Complementary searches (hand‐searching references in highly relevant articles including literature reviews, and specialist journals, for example, *International Journal of Artificial Intelligence*, *Journal of Medical Internet Research*, published after 2014) were conducted by the first author on the basis that hand‐searches can minimise the risk of bias in selecting literature for systematic reviews (Vassar, Atakpo, and Kash 2016).

After title and abstract screening of a total of 3617 articles retrieved from database searches, 239 studies were selected as potentially relevant for answering our review question and filtered against the inclusion and exclusion criteria after full‐text reading. More specifically, only articles with clear focus on mental health nursing (e.g., psychiatric care in hospital, inpatient or outpatient and community mental health nursing) were included. Similarly, only studies in which the use and purpose of AI were relevant to mental health nursing practice were considered. Literature studies in which the focus on mental health nursing practice and/or AI was lacking, unclear, or limited were excluded. Finally, articles that were not primary (e.g., secondary or tertiary research, commentaries, editorials, dissertations and conference proceedings) or published in a different language than English were excluded. To capture the state of art of using AI in mental health nursing, we only included recent literature published after 2014.

**FIGURE 1:**
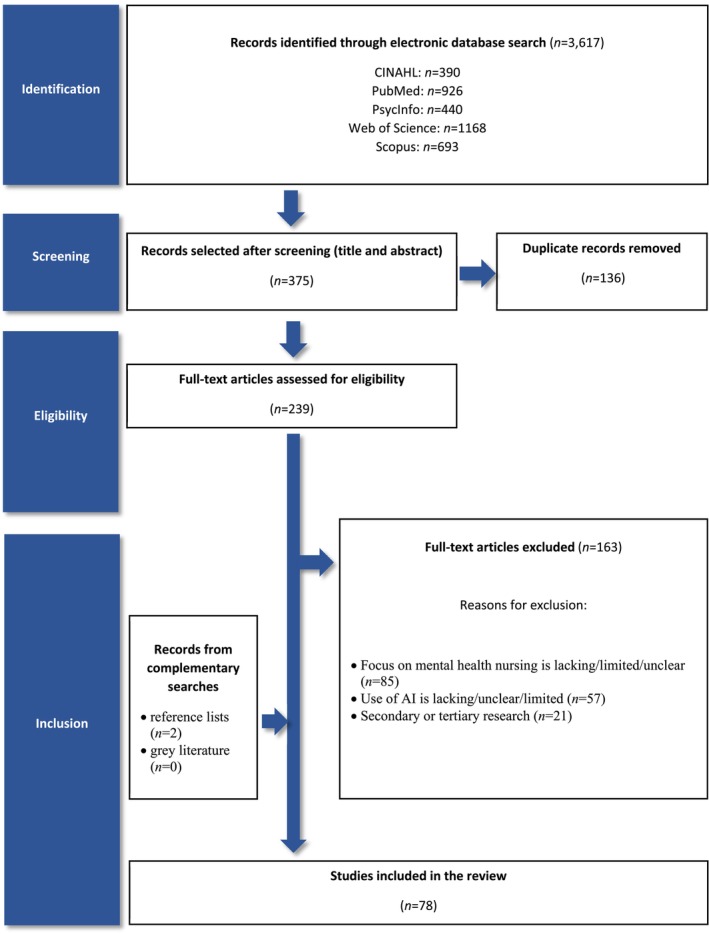
The literature search process represented in a PRISMA flow diagram (Page et al. 2020).

As a result of this process, we identified 78 studies (*n* = 76 from the electronic database searches and *n* = 2 from hand‐searching the reference lists of relevant literature reviews). We believe that the high number of studies on the use of AI with potential to enhance mental health nursing reflects the systematic process of literature searching adopted here (Gray 2020). An alternative explanation could be the exponential growth of literature on this topic as suggested in the results section and hinted at in other reviews (Thakkar, Gupta, and De Sousa 2024). The literature search process is illustrated in the PRISMA flowchart in Figure 1 adapted from Page et al. (2020).

The quality of the selected articles was appraised by the first author using the CASP (2018) checklist for qualitative, quantitative and mixed‐methods studies. No studies were excluded based on quality, but this aspect was reflected upon, and critical comments were included throughout the results section. A bespoke 13‐point data extraction tool was developed and piloted collaboratively by the authors including the aim and the context/setting of the study, research design, sample size, participants' characteristics, findings, and subsets of AI and their relevance to mental health nursing. This provided us with a base for the thematic data synthesis that followed the steps recommended by Thomas and Harden (2008): coding, clustering codes into descriptive themes, and amalgamating descriptive themes into analytical themes.

## 3 Results

### 3.1 Characteristics of the Studies

**FIGURE 2:**
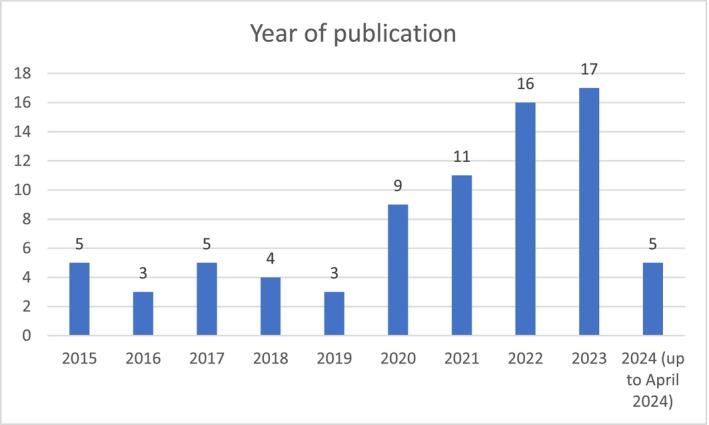
Year of publication of the selected articles.

From 78 articles included in this integrative review, 58 (72.5%) were published in the past 5 years. This trend (Figure 2) illustrates the exponential progression of research on this topic during and after the COVID‐19 pandemic that accelerated the implementation of remote care and digitalisation of nursing practice (Abdolkhani et al. 2022).

**FIGURE 3:**
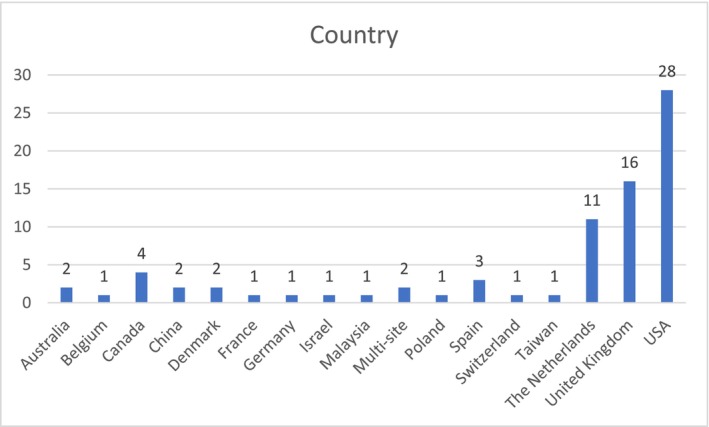
Countries in which the studies included in this review were conducted.

Geographically, the majority of studies (*n* = 55, 70.5%) originate from the United States (*n* = 28, 36%), the United Kingdom (*n* = 16, 20.5%) and the Netherlands (*n* = 11, 14%). The geographic profile of the research conducted on this topic (Figure 3) suggests that, with some exceptions, applications of AI in mental health nursing are not researched outside the developed countries. This may reflect some potential implications on global health inequities and varying levels of digitalisation of mental healthcare services internationally (Badr, Motulsky, and Denis 2024).

The selected literature is mostly quantitative (*n* = 74, 95%), although three mixed‐methods studies and one qualitative study were also identified with focus on capturing perceptions of mental health clinicians on adopting AI in practice. Quantitative research varied in design from experimental, quasi‐experimental, longitudinal, cross‐sectional and survey designs. The predominance of experimental studies indicates that this is an emerging area of research currently tested, piloted and validated before being widely implemented in practice.

In terms of quality, most of the literature complies with criteria for high‐quality research in terms of aim clarity, appropriateness of the research design and data collection and analysis methods, transparency of the recruitment process, presentation of the results and ethical considerations (CASP 2018). Limited generalisability was a recurrent issue in 33 articles (42%) with potential impact on the research findings. This was due mainly to small sample sizes in 11 articles (*n* < 100, average sample size 26 406), or the nature of the clinical setting that limits the translation of findings to other contexts.

### 3.2 Setting

Most of the studies (*n* = 56, 72%) were conducted in psychiatric hospitals covering a range of patients and electronic health records, e.g., paediatric, adolescents, adult, forensic and perinatal mental health, at different stages of hospitalisation from admission to discharge. A spectrum of mental health conditions has been identified, for example, depression, anxiety, bipolar disorder, borderline personality disorder, schizophrenia, psychosis, obsessive‐compulsive disorders and eating disorders. A smaller number of studies (*n* = 11, 14%) involved outpatients in community mental health settings, e.g., longitudinal studies of remission patterns and recovery processes over a longer period. Other settings (*n* = 6, 8%) included educational institutions and laboratories for experimental studies.

### 3.3 Types of AI

**FIGURE 4:**
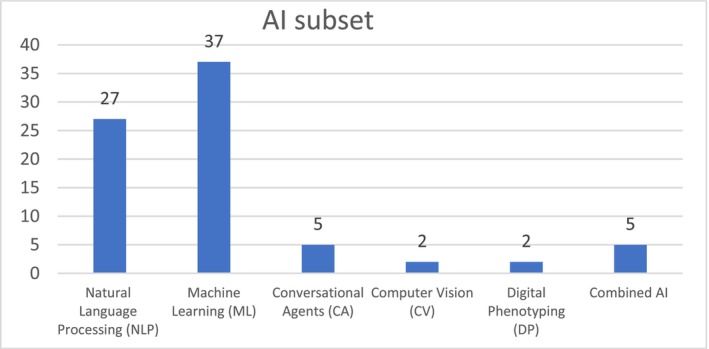
Subsets of AI utilised in the selected studies.

The main subsets of AI identified in our selected studies are machine learning (ML) (*n* = 37, 47%) and natural language processing (NLP) (*n* = 27, 35%) (Figure 4).

Machine learning (ML) enables systems to learn from data, for example, healthcare records, identify patterns and make decisions with minimal human intervention. It normally involves algorithms that improve automatically through experience (Habehh and Gohel 2021). An important subset of ML identified in this review is deep learning (DL) using neural networks to perform complex tasks like image and speech recognition.

Natural language processing (NLP) involves the interaction between computers and human spoken and written language. It includes tasks like text analysis, sentiment analysis and speech recognition, allowing machines to understand, interpret and generate human language (Hossain et al. 2023).

Less frequently, generative AI agents like ChatGPT are used alongside computer vision (CV) to interpret and understand digital images from cameras and videos, identify and classify objects, and make sense of the environment. A small number of studies used digital phenotyping (DP) to collect health data from digital devices to study and understand human behaviour and mental health.

Although these AI subsets are presented here as separate entities, they tend to overlap and are occasionally used in combination which was the case with five studies included in this review.

### 3.4 Themes

**FIGURE 5:**
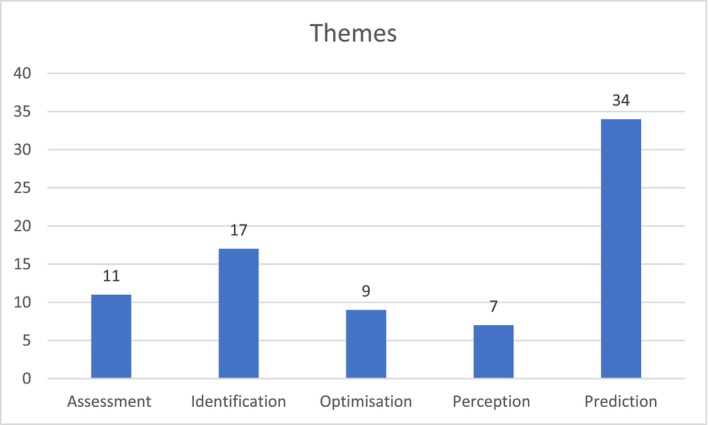
Themes identified following the thematic analysis of the data extracted from the selected articles.

Following the thematic synthesis (Thomas and Harden 2008), we identified five themes illustrating the potential of AI to enhance different areas of mental health nursing practice: assessment, identification, prediction, optimisation and perception (Figure 5).

#### 3.4.1 Assessment

Assessment in mental health nursing is a critical process involving gathering comprehensive information about patients' mental, emotional and physical health to understand their condition and provide appropriate care. The use of AI in the selected literature showed potential for enhancing this process by improving accuracy, efficiency, and personalisation of care.

For example, Bain et al. (2017) advance an AI platform (AiCure) using facial recognition to visually confirm ingestion of medication in schizophrenia patients via smartphone. Such techniques could allow mental health nurses to assess the degree of concordance with psychiatric medication more effectively considering its higher accuracy (89.7% vs. 71.9%) when compared to modified directly observed therapy, i.e., when a mental health nurse watches the patient swallow a portion of the prescribed drugs.

Another application of AI was identified in assessing the severity of mental distress in psychiatric patients. Huang et al. (2022) proposed a machine learning model to help automate the assessment of severity of thought disorder in schizophrenia by processing textual and acoustic speech between psychiatric nurses and schizophrenia patients. The preliminary results of this study are promising in terms of potentially addressing issues of imbalanced ratio between trained mental health professionals and patients with schizophrenia in Taiwan, once the model is validated in real‐world scenarios. Similarly, Posada et al. (2017) successfully assessed symptom severity from initial psychiatric evaluation records using NLP techniques and predictive modelling with detection of subtle diagnostic differences.

Particular attention seems to be given to using AI solutions for assessing risk behaviours. Levkovich and Elyoseph (2023) compared the assessment of suicide risk carried out using ChatGPT‐4 with routine assessments conducted by mental health professionals. They concluded that the evaluation of likelihood of suicide attempts in psychiatric patients evaluated by ChatGPT‐4 was similar to that performed by mental health professionals. This seems to be an improvement compared with previous versions of ChatGPT‐3.5 showing a tendency to underestimate the potential for suicide attempts when compared to assessments conducted by trained mental health professionals.

AI algorithms go as far as (in)validating the performance of suicide‐related assessment instruments used routinely in mental health practice. Edgcomb et al. (2023) tested a ML‐based model that significantly improved the sensitivity of detecting suicide cases in children presenting with self‐injurious thoughts and behaviours. Such results indicate the possibility of applying ML approaches to standard assessment tools and codified mental health records to reduce cases of missing patients with suicidal ideation and behaviours.

A different AI subset, namely, data mining of electronic health records through natural language processing (NLP), was used by Castro et al. (2015) to train a diagnostic algorithm for assessing individuals with bipolar disorder. The outcomes were comparable to gold‐standard diagnostic interviews illustrating the potential of AI‐driven approaches to learn to distinguish between different experiences of distress by assimilating high volumes of health data. This is supported by Barroilhet et al.'s (2020) study using NLP to assess personality features in psychiatric patients. The clinical implications of such advancements are unlimited if we consider AI subsets like computer vision able to estimate the severity symptoms of schizophrenia by assessing facial expressions of psychiatric patients (Bishay et al. 2021).

Furthermore, AI capabilities have expanded to learning about psychiatric diagnostic categories by analysing patients' lived experiences and self‐reported symptoms with ML techniques (Ghosh et al. 2022). Interestingly, the AI algorithm in Gosh et al.'s (2022) study learning from patients' experiences of distress generated four categories of mental health challenges significantly less than conventional approaches like DSM‐5 relying more on intuition and consensus than data which makes the current diagnostic systems questionable in terms of bias, heterogeneity, reliability and validity.

These developments certainly raise some questions whether assessing mental health conditions is indeed reserved exclusively to humans when ML and other subcategories of AI perform as well, or even more effectively, compared to mental health professionals. Some studies included in this review add to this debate by demonstrating issues with AI performing poorly when attempting to discern sentiment from session records in patients with eating disorders when compared to human raters (Huisman, Kraiss, and Alexander de Vos 2024).

Our findings indicate that AI‐driven mental health assessments, despite showing potential in terms of accuracy, cannot be implemented in mental health nursing practice in their current state without addressing ethical and procedural complexities explored in more depth in the discussion section.

#### 3.4.2 Identification

Identifying mental health symptoms, behaviours and risk factors is a process closely related to assessment. While assessing involves a comprehensive and ongoing process consisting of gathering, analysing and interpreting data about a patient's mental health status, identification refers to highlighting specific issues or patterns that require attention drawing from health records. Due to the unstructured nature of health data partly caused by the fragmentation of healthcare provision, it is almost impossible for mental health nurses to process high volumes of information to detect trends and patterns that could potentially inform their practice.

AI‐driven solutions known for processing large datasets address such limitations as illustrated in studies drawing on healthcare data to identify risk issues critical in mental health nursing practice such as suicide (Bohaterewicz et al. 2021; Carson et al. 2019; Fernandes et al. 2018), self‐harm (Ayre et al. 2021) and substance misuse in psychiatric patients (Afshar et al. 2019; Carrell et al. 2015; To et al. 2020).

For example, Afshar et al. (2019) analysed 91 045 electronic health records of trauma patients combining NLP and ML techniques identifying alcohol misuse with an accuracy of 0.78. Similarly, To et al. (2020) developed an alcohol misuse classifier using NLP validated in a cohort of 1000 hospitalised patients performing at even higher level (0.91). Although yet to be generalised through larger scale studies, the results are promising for expanding such tools to provide standardised methods for identifying substance misuse in psychiatric care.

This is also the case with studies focused on identifying risk behaviours such as suicidal ideation and self‐harm. Bohaterewicz et al. (2021) combined ML algorithms with resting‐state functional magnetic resonance imaging to differentiate schizophrenia patients at risk of suicidal risk. However, the small sample size (*n* = 59) warrants caution in generalising the results. Good performance (82.8% precision) of identifying suicidal ideation and attempts from electronic health records of psychiatric patients was also reported by Fernandes et al. (2018) who employed a NLP techniques. The latter study also emphasises the adaptability of AI algorithms to fit the infrastructure of different clinical datasets which is promising for generalising AI solutions beyond the experimental settings. Finally, a more modest rate (0.47 accuracy) was the result of a ML approach to analysing electronic health records of adolescents hospitalised in a psychiatric inpatient unit (*n* = 73). Although in its infancy, the process of identifying risk behaviours may be an important step in suicide prevention practice and enhancing safety and quality of care provided by mental health nurses.

Free‐text health records frequently used in psychiatric care appear to be an invaluable source of information for identifying other aspects critical to mental health nursing practice: for instance, identifying adverse drug events like extrapyramidal effects of antipsychotic therapy with a precision of 0.85 in a diverse cohort with different diagnoses, gender, age, and ethnicity (Iqbal et al. 2015). Similarly, Kadra et al. (2015) advanced a novel method relying on NLP techniques to identify instances of antipsychotic polypharmacy. Kadra et al.'s (2015) study stands out with a large database of 230 000 patients with severe mental illness and high precision of identifying co‐prescribing antipsychotic medication (0.94–0.97). As such trends are difficult to recognise in clinical practice without the aid of AI, the findings are promising for valuable feedback to inform prescribing guidelines in mental health nursing practice.

This theme is also informed by interesting findings from forensic psychiatry represented by studies focused on the identification of psychiatric patients at risk of committing crime (Trinhammer et al. 2022) including paedophilic offenders (Popovic et al. 2023) through data mining of patient registries via ML algorithms. Although the accuracy rates are modest (57% and 75.5%, respectively), the findings open opportunities for identifying potential future forensic psychiatric patients allowing for early‐intervention initiatives in this clinical area.

#### 3.4.3 Prediction

Prediction refers to anticipating future mental health issues, behaviours or outcomes based on current data and trends identified in mental healthcare records. Prediction involves prognostic and forecasting of mental health conditions, for example, relapse or recovery outcomes and treatment trajectories. AI‐driven predictive algorithms in mental health are key for initiating preventative measures, planning treatment and tailoring interventions to minimising risk, and improving long‐term mental health outcomes.

A cluster of studies within this theme is focused on predicting recovery or remission in patients with various mental health conditions such as depression (Benoit et al. 2022; Chekroud et al. 2016; Dinga et al. 2018; Salem et al. 2023), anxiety (Bokma et al. 2022), psychosis (de Nijs et al. 2021), eating disorders (Haynos et al. 2021) and OCD (Grassi et al. 2022). Interestingly, the recovery prediction rates vary from higher levels when recovery is conceptualised as remission of symptoms through pharmacological treatments, to more modest levels when psycho‐social factors are considered. The latter reiterates the complexity and nonlinear nature of recovery from mental distress that appears to pose challenges to AI algorithms, particularly when long‐term recovery is considered.

Contrastingly, other studies are centred on predicting relapse in obsessive‐compulsive disorders (Askland et al. 2015) and peri‐natal mental health (Khapre, Stewart, and Taylor 2021). To complement this, the predicting re‐admission or re‐hospitalisation of psychiatric patients along with future service consumption was the focus of studies conducted by Ab Kader, Yusof, and Khald (2023), Blankers, van der Post, and Dekker (2020), Boag et al. (2021), Góngora Alonso et al. (2022), Silva et al. (2021) and Van Mens et al. (2023). While the prediction rates vary considerably among these studies, it is clear that the unpredictability of mental health difficulties makes the predictions challenging, especially when the patient healthcare data are limited or when patients have co‐existing psychiatric conditions.

Some studies produced ML‐based models to predict remission in patients undertaking psychiatric treatments such as desvenlafaxine (Benoit et al. 2022) or escitalopram (Chekroud et al. 2016) with an accuracy of 0.69 and 0.59, respectively. Other authors advance models for predicting the satisfaction of patients with psychiatric medication with an accuracy rate of 0.87 (Wang et al. 2023). Although the models' performance is above chance, they require large‐scale testing and validation before implementing them into practice to enable prospective identification of patients who are likely to respond to a specific treatment. With regard to potential clinical implications, Benoit et al. (2022) claim that ML models of treatment prediction can influence mental health practice by (1) identifying which patients should (not) receive certain psychiatric treatments and (2) allowing clinicians to compare remission probabilities of multiple drugs and identify best routes of treatment (including factors such as cost and potential side‐effects).

As with the previous themes focused on assessing and identifying risk behaviours, prediction of suicidal risk was central to Barrigon et al. (2023), Cook et al. (2016), Liberman et al. (2022) and Papini et al. (2024). The prediction rates in these studies varied between 0.73 and 0.92. However, the findings are difficult to compare due to different AI subsets and methodologies being used by researchers, for example, ML based on smartphone data and NLP of hospital discharge records. Papini et al. (2024) claim that predicting suicidality with AI‐driven models is more accurate than traditional statistical models with significant implications for public health, suicide prevention, monitoring and providing early intervention for high‐risk groups. However, more work is underway to incorporate data collected from patients and caregivers alongside clinical notes to improve suicide risk detection (Barrigon et al. 2023).

More uncommon but nonetheless relevant to mental health nursing practice, other risk factors and patient behaviours, e.g., aggression, are the focus of Danielsen et al.'s (2019) study in which ML algorithms were trained to predict mechanical restraint of psychiatric inpatients drawing on their electronic health data. Their prediction rate (0.87) was higher than that in Magnowski et al.'s (2022) study in which the need for restraint and seclusion were correctly predicted in 73% of psychiatric at‐risk patients. The findings are complemented by prediction of prospective incidents of physical aggression in psychiatric patients (Watts et al. 2023), indicating potential to develop individualised patient treatment plans to facilitate a proactive approach to preventing behavioural emergencies requiring restraint and seclusion (Magnowski et al. 2022). Such solutions hold promise for enhancing quality care and patient and staff safety in inpatient mental health services, along with improved monitoring and timely interventions.

#### 3.4.4 Optimisation

The number of studies exploring optimisation of mental health and psychiatric nursing practice through AI technologies was significantly lower compared to other themes. However, it can be claimed that effective assessment, identification and prediction of various aspects of mental healthcare could potentially lead to service optimisation. An alternative explanation can be found in the predominance of experimental studies yet to be validated and generalised in real‐life contexts to create impact on practice and, subsequently, on optimising mental healthcare.

While the assessment, identification and prediction themes are mostly focused on individuals with various mental health conditions, optimisation is presented here at service level starting with personalising mental health referral pathways with AI‐powered chatbots (Habicht et al. 2024), optimising waiting times (Rastpour and McGregor 2022) and optimising practitioners' use of time (Rollwage et al. 2024). Furthermore, ensuring effective allocation of psychiatric beds (Hudson 2021), providing telepresence alternatives for isolation wards (Wang et al. 2023), and potentially replacing in‐person nursing observations in mental health inpatient wards with AI assistants (Barrera et al. 2021) are other areas in which AI can optimise mental health nursing practice.

Moreover, a study conducted by Shah et al. (2022) evaluated the usability of a chatbot prototype for promoting mental health utilisation by improving eating disorder service users' motivation for treatment and self‐efficacy. Such solutions positively received by individuals with eating disorders can supplement mental health nurses' role in psycho‐education, conducting motivational interviewing and personalising psychiatric treatments.

Finally, Cousins et al. (2022) employed neural networks to optimise treatments for depression using data from specialist and community psychiatric services in Australia, New Zealand, and Japan. The estimated result of this initiative is the reduction of treatment optimisation time for patients suffering with depression potentially leading to a faster recovery. From a service perspective, introducing web‐based tools for remote monitoring and ML algorithms may assist mental health services with extending their remit to larger patient communities (Cousins et al. 2022). Coupled with findings from Knights et al. (2023) advancing AI solutions for ensuring optimal levels of mental healthcare are available at the right time for patients, the optimisation of mental health services through AI becomes more achievable and necessary.

Although studies directly addressing this area remain sparse, the implications of AI‐enabled optimisation on service delivery are nonetheless significant. For example, by streamlining referrals, AI tools can reduce inefficiencies and service gaps, improve accessibility and enhance patient outcomes. It can also result in alleviating some of the administrative burden on mental health nurses who can reinforce the human element of care focused on compassionate care and individualised support.

#### 3.4.5 Perception

Our review captured a myriad of AI‐powered technologies with potential to enhance different aspects of mental healthcare particularly in psychiatric settings. However, it is essential to understand how mental health nurses and other professionals perceive such innovations capable of changing and challenging more traditional ways to deliver mental health services.

Several studies have been conducted to investigate perspectives of mental health nurses and other clinicians, nursing students, and patients and carers on the use and acceptance of AI in clinical practice. For instance, Blease, Worthen, and Torous (2024) reported that 4 in 10 mental health professionals use ChatGPT to assist in answering clinical questions leading to documentation efficiency and decreased administrative burden. As a result, the input of generative AI tools in psychiatric care is inevitable holding ‘tremendous potential to augment our profession’, as expressed by a participant in this study. However, Blease, Worthen, and Torous (2024) also highlighted concerns around the potential harm from AI applications perceived as less reliable and biased, e.g., structural racism in mental healthcare.

Another study conducted by Brown et al. (2020) evaluated clinicians' perceptions of suicide risk flags generated with ML algorithms. Most of the clinicians surveyed in this study reported suicide risk flags in electronic medical records would significantly alter their clinical decision making. However, 94.12% of the respondents showed a preference to knowing which patient characteristics led to a suicide flag, while 88.24% reported that knowing these characteristics would influence their plan of action. These figures suggest that clinicians do not blindly rely on decisions made by AI‐driven technologies and require information for decision making beyond the ‘black box’ of AI algorithms. A similar topic was explored form patients' perspective in a qualitative study conducted by Yarborough et al. (2022). The general view of participants in this study was supportive of suicide risk prediction models derived from electronic health data with some reservations concerning the risk of AI to induce anxiety or trigger coercive treatment. Also supportive was the attitude of adolescent patients and their parents towards digital phenotyping, i.e., use of data collected through digital devices such as smartphones and wearables, to measure, analyse and understand health behaviours and experiences (Orr et al. 2023). However, the more detailed the information collected, the lower the level of acceptance in this study which indicates that digital phenotyping may be perceived to be intrusive to a certain extent.

More scepticism towards AI is evidenced in Zhang et al.'s (2023) study exploring mental healthcare professionals' perspectives on adopting AI in clinical practice. Interestingly, mental health nurses in this study expressed a lower level of comfort with AI‐based clinical applications compared to educators, researchers, and other clinicians. A possible explanation offered by the authors is the limited knowledge and awareness of AI in mental health nursing. The study also reveals a level of uncertainty around merging humanistic approaches to mental healthcare with AI solutions risking to negatively impact on the therapeutic relationship with patients. As a result, Zhang et al. (2023) noted a tone of resistance and negativity in mental health practitioners towards implementing AI solutions in practice, doubled by their fear of compromising the quality of care. Reservations towards using digital phenotyping technologies were also expressed in Bourla et al.'s (2020) study conducted with mental health nursing students in relation to AI's reliability and its potential to hinder the therapeutic relationships. However, the overall acceptability of such approaches to healthcare in this study was overall high, which may suggest more openness towards accepting AI‐driven solutions from future generation of nurses (74.5% aged below 25 years).

Interestingly, only a few studies included in this review touched upon ethical concerns around using AI in mental healthcare. A mixed‐methods study exploring consumer perspectives on AI technology and automation in crisis support services (Ma et al. 2022) captured ethical issues more comprehensively. In Ma et al.'s (2022) study, privacy and data sharing issues are discussed from the perspective of service users particularly in relation to data potentially being used against them in the future, and the limited control over the information being shared. The lack of discussions around ethical considerations may be due to the experimental nature of studies included in this review. As a result, although ethical approval was required, AI‐based mental health studies are still in the early stages of implementation with ethical issues still to emerge.

## 4 Discussion

The aim of this review was to comprehensively identify and synthesise current evidence of AI‐driven applications with relevance for, and potential to enhance, mental health nursing practice. We addressed this aim by drawing on 78 relevant articles selected through a systematic and rigorous methodology. The literature review process revealed five themes, i.e., assessment, identification, prediction, optimisation, and perception, discussed in this section with reference to the wider literature and emphasis on implications for the future of mental health nursing practice.

### 4.1 Assessment

Assessment is an essential competence in mental health nursing. Mental health risk assessments are particularly important to protect patients and others from harm. However, mental health nurses experience difficulties with identifying patients at risk, one reason being the conflict between their instinct and actuarial data when making clinical decisions (Conlon, Raeburn, and Wand 2024). With the increasing digitalisation of care, it is only a matter of time until AI will aid mental health nurses' routine assessments with a rigorous analysis of healthcare data.

In the context of a mental healthcare system within which suicide prevention is in need for improvement following the ever‐increasing rates of deaths, suicide attempts, and self‐harm (World Health Organisation 2023b), AI‐assisted assessments and screening could provide timely and cost‐effective solutions. Kammer et al. (2021) claim that knowing which services are accessed most by at‐risk individuals before a suicide attempt or self‐harm can inform suicide prevention strategies. However, due to the unstructured nature of health data and fragmentation of health provision, this task is beyond human capabilities. Implementing AI‐based solutions shown to autonomously learn at incredible rates from high volumes of data could address mental health nurses' limitations in monitoring and assessing patients' complex journey through the healthcare system. This includes adherence to psychiatric treatments linked to increased hospitalisation rates (Karve et al. 2014) and relapse (Caqueo‐Urízar et al. 2021) that can be assessed effectively with AI (Bain et al. 2017), as demonstrated in other clinical areas (Babel et al. 2021).

The integration of AI into mental health nursing holds transformative potential, offering enhanced accuracy, efficiency and personalisation in patient assessments. AI technologies for evaluating medication adherence, for example, AiCure® (Bain et al. 2017), have shown significantly higher accuracy than traditional methods, suggesting that nurses could rely on these tools to improve treatment outcomes. Similarly, machine learning models that assess the severity of mental health conditions or suicide risks offer solutions to workforce shortages by automating routine assessments, allowing nurses to focus on complex care needs (Rony, Parvin, and Ferdousi 2024).

### 4.2 Identification

The digitalisation of healthcare services and increasing volume and complexity of electronic health records allow for AI algorithm development by deep mining the ‘big data’ with which AI is known to have a synergistic relationship (Agrawal and Prabakaran 2020). As a result, AI can be trained to identify issues and patterns to inform mental healthcare provision. Identification of risk behaviours, as with their assessment, is an important step in initiating preventative measures in mental healthcare. This task can be performed by AI platforms with varied degrees of success, as illustrated by our findings (Bohaterewicz et al. 2021; Fernandes et al. 2018). A review conducted by Rony, Parvin, and Ferdousi (2024) concluded that pattern identification following an analysis of vast amounts of patient data aided by AI systems holds potential for improved patient safety and nursing efficiency. For example, equipping nurses with real‐time alerts allows for planning timely interventions and preventative strategies (Olawade et al. 2024).

However, the use of AI has been expanded beyond detecting risk behaviours. Our findings suggest a more nuanced picture with a range of applications in mental health nursing including identification of issues with administering psychiatric medication (Iqbal et al. 2015; Kadra et al. 2015). Polypharmacy and adverse drug events are known to be critical issues in mental healthcare with negative impact on patients' quality of life and adherence (Jerjes et al. 2024). Despite efforts to reduce the incidence of polypharmacy, it remains highly prevalent in psychiatric patients leading to lengthier hospital stays and increased healthcare costs (Carmona‐Huerta et al. 2019). AI has been identified in this review as a potential solution to personalise prescription of psychiatric medication, provide useful information for ‘de‐prescribing’ unnecessary medications and reduce the risks of adverse drug events, the ‘pill burden’ and financial costs in psychiatric patients (Halli‐Tierney, Scarbrough, and Carroll 2019). Similarly, AI has been shown to facilitate the prevention of medication errors and improve medication management in other clinical settings (Damiani et al. 2023; Zheng et al. 2023). This aspect is highly relevant in the context of medication errors remaining a common risk to patient safety in psychiatric hospitals (Keers et al. 2018).

AI‐driven solutions hold promise for transforming mental health nursing practice in what concerns identifying critical symptoms, behaviours, treatment responses and risk factors. The unstructured nature of health records often hampers the ability of nurses to process large datasets effectively, but AI techniques like natural language processing (NLP) and machine learning (ML) can address these challenges, as demonstrated in other areas of nursing (Ng et al. 2022). Additionally, a more accurate identification of key issues to patients' mental distress and recovery may lead to personalised care plans reflecting their needs and recovery goals and outcomes instead of prioritising organisational agendas (Brooks et al. 2018).

### 4.3 Prediction

Assessing and identifying mental health issues in clinical practice are complemented by predictive AI algorithms with potential to forecast the course of psychiatric conditions, for example, recovery or relapse (Benoit et al. 2022; Bokma et al. 2022; Chekroud et al. 2016; de Nijs et al. 2021; Dinga et al. 2018; Grassi et al. 2022; Haynos et al. 2021; Salem et al. 2023). Electronic healthcare records may hold the key for predicting re‐hospitalisation of psychiatric patients (Kader, Yusof, and Khald 2023; Blankers, van der Post, and Dekker 2020; Boag et al. 2021; Góngora Alonso et al. 2022; Silva et al. 2021, and Van Mens et al. 2023), although the prediction rates vary widely. The possibility to predict mental healthcare consumption via AI opens new avenues for enhancing person‐centred care while contributing to more efficiency in service delivery. This aspect is essential considering the ever‐growing demand for mental health services alongside increasing complexity of mental health conditions (Sabella and Fay‐Hillier 2014; Cranage and Foster 2022).

However, the predictive potential of AI clashes with the unpredictability of mental health issues that, unlike many physical health conditions, can fluctuate widely influenced by a range of biological and psychosocial factors, as shown in a study of borderline personality disorder (Mneimne et al. 2021). This may explain why predictive algorithms in the selected studies are frequently focused on extreme behaviours such as suicidal attempts and self‐harm more evident from patients' health records (Barrigon et al. 2023; Cook et al. 2016; Liberman et al. 2022, and Papini et al. 2024). Our claim is supported by differences between the accuracy rates of predicting suicidal behaviours noted in the selected studies (0.73–0.92) and prediction rates of remission in patients undertaking psychiatric treatments (0.59–0.69). The later might be more difficult to predict due to high degrees of variability in responding to psychotropic medication (Radua, Davies, and Fusar‐Poli 2021). Also, it is known that recording data on risk behaviours takes priority in mental health nursing practice (Conlon, Raeburn, and Wand 2024) potentially resulting in more detailed information on this aspect that can be processed and analysed by AI algorithms. Furthermore, routine assessment and recording of risk may involve standardised instruments more accessible to AI interpretation compared with narrative notes (Coats et al. 2020). However, our findings indicate increasing use of natural language processing (NLP) to extract meaningful data from clinical free‐text notes to make predictions that could potentially assist mental health nurses in providing better patient care while supporting their decision making (Mitha et al. 2023).

The impact of AI‐driven predictive algorithms in mental health nursing and beyond can have significant implications for enhancing proactive, personalised, and preventative care while allowing for timely interventions. For instance, algorithms predicting recovery from conditions like depression or anxiety can help nurses adjust care plans and support preventative measures to reduce hospital readmissions and improve long‐term outcomes. Personalised treatments can also be enhanced through AI algorithms predicting which interventions patients are most likely to be satisfied with and succeed as treatment avenues. Corroborating the predictive power of AI with nurses' qualitative insights and assessment of patients' psycho‐social factors could be the key to preserving the human component of care in this profession while capitalising on AI's potential (Yelne et al. 2023).

### 4.4 Optimisation

Mental health nursing worldwide is facing numerous challenges such as the ever‐growing demand for mental health services and limited resources leading to budget cutbacks and downsizing services. This has a significant impact on the quality of care provided, and the increasing psychosocial complexity of the mental health nurses' role beyond the traditional bio‐medical paradigm (Cranage and Foster 2022; Elliott and Masters 2009; Sabella and Fay‐Hillier 2014). Such issues have been exacerbated by the COVID‐19 pandemic that stretched mental health nursing workforce with long‐term social, psychological, and moral implications (Frawley et al. 2020). As a result, there is a call for reforming the nursing sector worldwide for a more effective utilisation of the potential and capabilities of mental health nurses (Hurley et al. 2022).

A digital reformation of the nursing sector is already taking place including the implementation of AI‐driven tools to support care and enhance nurses' understanding of, and potential to address, complex health issues (O'Connor, Devane, and Rose 2023). As a result, the use of AI holds promise to improve clinical efficiency by reducing the time mental health practitioners spend on assessments by 23%, as estimated by Rollwage et al.'s (2024). Such outcomes are crucial considering that time spent with patients is a key factor in mental health nursing practice. Glantz, Sunnqvist, and Örmon (2023) evidenced this aspect using a time‐geographic perspective revealing that nurses in psychiatric inpatient care spend less time with patients than desired, and they tend to spend little time in places where patients are. This may be because 70% of a clinician's time is dedicated to administrative work when 44% of clinical administrative tasks can be automated or reassigned to AI (Willis et al. 2020). Therefore, AI can help mental health nurses free a significant amount of administrative time that can be invested more meaningfully in direct interactions with their patients (Woodnutt et al. 2023) potentially benefiting the development of therapeutic relationships key to successful delivery of mental healthcare (Bacha, Hanley, and Winter 2020).

Another example of service optimisation is provided by Rastpour and McGregor (2022) who used machine learning for a comprehensive analysis and optimisation of the waiting times for new psychiatric patients. Although the generalisability of this study is limited due to the small sample size, it shows potential to address a critical issue in mental healthcare. Waiting times are associated with a range of negative psychological and behavioural implications that often contribute to further deterioration of individuals' mental health (Punton, Dodd, and McNeill 2022; Reichert and Jacobs 2018). Coupled with a more effective allocation of psychiatric beds utilising ML algorithms (Hudson 2021), it may lead to an improved patient flow in psychiatric wards (Turkington et al. 2020).

AI technologies appear to be the key for optimising mental health nursing by improving efficiency, decision making, and patient care. Optimising waiting times, streamlining referrals, and reducing administrative tasks allow nurses to focus on compassionate and individualised care, therefore reinforcing their humanistic role in delivering mental healthcare. Such aspects could also support the ‘balancing act’ between human and AI collaboration that continues to be at the centre of controversies around implementing AI solutions in healthcare (Welch et al. 2024).

### 4.5 Perception

The benefits of implementing AI platforms in mental health nursing are evident. However, there is little evidence with regard to patients' and staff's perspective on implementing AI solutions in mental healthcare. The few studies included in this review paint a mixed picture of how AI is perceived by mental health clinicians and patients although attitudes may be well influenced by the social‐cultural and organisational context of the research (Barnes, Zhang, and Valenzuela 2024).

On the one hand, AI solutions are welcomed by those involved in the act of care due to their cost‐ and resource‐effectiveness, timeliness, and potential for enhanced decision making. The openness towards novel AI technologies and recognition of their benefits in nursing have been captured in other reviews of attitudes of patients and carers (Fritsch et al. 2022) and staff (Sommer, Schmidbauer, and Wahl 2024).

On the other hand, resistance to using AI has been identified in our selection of articles supported by Robertson et al. (2023) who noted a higher degree of reluctance in old people and individuals from Black communities. This may reflect patients' preference for human health professionals to AI‐driven applications in assessment, diagnosis, and treatment (Longoni, Bonezzi, and Morewedge 2019; Young et al. 2021). We identified a level of resistance in what concerns mental health nurses and other clinicians fuelled mainly by the limited knowledge and awareness of AI in practice. This is understandable considering the inherent humanistic formation of mental health nurses working with subjective experiences difficult to envisage as a domain of machine learning. Zhao (2023) refers to this context as ‘post‐human era’ or ‘technology‐integrated ecosystem’ in nursing in which the boundaries between humans and machines are increasingly blurred. A potential solution to this ideological conflict is the promotion of a human–machine partnership or co‐creation instead of envisaging a future in which nurses' jobs are endangered by AI (Vasquez et al. 2023).

Considering the fast‐paced advancement of AI technologies, there is an urgent need to train and educate the nursing workforce for a more comprehensive understanding of AI principles and applicability to clinical practice (Abuzaid, Elshami, and Fadden 2022) and education (Glauberman et al. 2023). On this note, our review may be informative to policy development in embedding AI technologies in mental healthcare prioritised in the NHS Long‐Term Workforce Plan (NHS England 2023). Additionally, ethical issues around incorporating AI in mental health nursing require further exploration particularly when AI technologies transition from theoretical models and prototypes to practical applications. This aspect appears to be underresearched in the studies reviewed here, in contrast with the wider literature pointing at ethical concerns in this area. Loch et al. (2022) draw attention to the impossibility to interpret ML algorithms and fully understand their implications for decision making due to their ‘black box’ models in which variables and the way they are analysed are not entirely observable. Consequently, it is almost impossible to assess the risk of hidden bias, for example, social and gender bias, inequities, and structural racism, which is evidenced in textual information like patient notes (Parikh, Teeple, and Navathe 2019; Sun et al. 2022). Moreover, issues of privacy, security and confidentiality have been raised by Nashwan and Abujaber (2023) in the context of AI processing high volumes of sensitive patient data, suggesting the need for clear policies and guidelines to ensure ethical integration of AI in healthcare.

Finally, the involvement of patients and mental health nurses in developing AI platforms appears to be limited, which may explain their resistance towards technology. This is the case in other areas of digital health, for example, telehealth, with regard to personal security concerns (Cook et al. 2016). Therefore, a strategic approach involving patient and staff more actively in the development of AI solutions is required. For instance, Kormilitzin et al. (2023) advocate for participatory approaches to AI development including LGBTQ+ communities to cater for potential bias that AI algorithms can carry. This falls under the paradigm of design justice ensuring representativeness of the end‐point users of AI technologies (Zidaru, Morrow, and Stockley 2021).

### 4.6 Strengths and Limitations

To our knowledge, this is the most comprehensive review up to date with a focus on the potential of current AI developments to enhance mental health nursing practice. The review was conducted by a team of two reviewers to increase the number of relevant studies and reduce selection bias (Stoll et al. 2019). The methodology for conducing the literature search, data collection, extraction and analysis proposed here is transparent and highly replicable for future iterations of this review, guided by a protocol published on PROSPERO (Ref. CRD42021234843) and the PRISMA guidelines (Page et al. 2020). The inclusion of multiple research designs allowed an in‐depth investigation of the topic also capturing the perspectives of mental health patients and professionals on the implementation of AI in clinical practice.

Several limitations must be acknowledged when interpreting the review findings. First, the language of the selected articles was limited to English, resulting in potentially omitting relevant articles from Asian countries under‐represented in this review with tradition in implementing and researching AI‐based healthcare technologies. Second, the search strategy could have been refined further with other relevant keywords, for example, ‘smart home’ or ‘ambient assisted living’ to capture a wider variety of AI applications in community settings. Finally, distinguishing between nursing and other types of mental healthcare was challenging at times due to overlaps and multidisciplinarity in clinical practice and research, for example, psychiatry, clinical psychology, and psychotherapy. As a result, research from other areas of mental healthcare potentially relevant to mental health nursing was excluded from this review. Therefore, we suggest that separate reviews are conducted to investigate the applicability of AI‐based technologies in other fields of nursing practice (e.g., children, learning disabilities, and dementia) for cross‐fertilisation of knowledge.

## 5 Conclusion

This integrative review comprehensively identified and synthesised the evidence on AI‐based applications with relevance for, and potential to enhance, mental health nursing practice. The findings suggest a nuanced picture of AI‐driven applications in mental healthcare that could potentially shape the future of mental health nursing profession. Although most of the studies included in this review are at the early stages of implementation, it is only a matter of time until AI will influence mental health nurses' clinical practice. This appears to take place at multiple levels including assessment and identification of critical mental health issues from electronic health records and predicting the course of mental health conditions and treatments. Furthermore, AI holds potential to optimise services by addressing current challenges in mental health nursing such as ever‐increasing demand for mental healthcare, limited resources, and long waiting times. While the benefits of AI for mental health nursing are multifold, so are the ethical concerns around implementing AI solutions in clinical practice discussed in this article. The success of addressing such challenges depends on the active involvement of patients and mental health nurses in developing future AI platforms.

## 6 Relevance for Clinical Practice

The article sheds light on the future of the mental health nursing profession as painted by the current evidence on the impact AI technologies may have on improving clinical areas such as risk assessments, predicting psychiatric diagnoses and the course of mental health conditions and treatments, alongside other AI‐driven approaches to optimising mental healthcare. Issues around preservation of humanistic and person‐centred care are discussed from an ethical perspective alongside with recommendations for collaborative practice in developing AI solutions in mental health nursing.

## Author Contributions

The first author conceptualised the research question, designing the methodology, and producing the review protocol (PROSPERO CRD42021234843). The literature search strategy was piloted by the first and second authors on CINAHL alongside screening the titles and abstracts of potentially relevant studies across five electronic databases. The quality of the selected studies and extraction of data were completed by the first author who drafted the initial manuscript and coordinated the overall review process with feedback from the second author.

## Ethics Statement

The authors have nothing to report.

## Conflicts of Interest

The authors declare no conflicts of interest.

## Data Availability

The data that support the findings of this study are available from the corresponding author upon reasonable request.
